# Advances in Applications of Polysaccharides and Polysaccharide-Based Materials

**DOI:** 10.3390/ijms25126482

**Published:** 2024-06-12

**Authors:** Sankarprasad Bhuniya, Tatiana S. Demina, Tatiana A. Akopova

**Affiliations:** 1 Centre for Interdisciplinary Sciences, JIS Institute of Advanced Studies and Research, JIS University, Kokata 700091, India; spbhuniya@jisiasr.org; 2 World-Class Research Center “Digital Biodesign and Personalized Healthcare”, Sechenov First Moscow State Medical University, 8-2 Trubetskaya Str., Moscow 119991, Russia; demina_t_s@staff.sechenov.ru; 3 Enikolopov Institute of Synthetic Polymeric Materials, Russian Academy of Sciences, 70 Profsoyuznaya St., Moscow 117393, Russia

Polysaccharides, complex carbohydrates composed of long chains of residues of sugar molecules, have garnered significant attention in recent years due to their diverse applications across various industries. From food and pharmaceuticals to materials science and biotechnology, polysaccharides and polysaccharide-based materials offer versatile solutions to modern challenges. This essay explores the recent advancements in the applications of polysaccharides and their derivatives, highlighting their role in driving innovation and sustainability.

Polysaccharides serve as the foundational components of genetic materials and as a primary energy source. They are essential constituents of living cells, participating in vital cellular and intracellular processes; of cell surface receptors; of signaling molecules; and of bacterial adhesives. These multifaceted roles underscore the potential of carbohydrates as pharmaceutical and diagnostic agents, driving synthetic chemists to explore various methods for synthesizing diverse glycoconjugates. Numerous carbohydrates, both natural and synthetically derived, find clinical utility in treating various diseases. Their structural diversity, characterized by differing functional groups, linkages, and ring configurations, renders them invaluable for designing and developing biologically active glycoconjugates. Following thorough chemical and biological investigations, carbohydrate-based entities have emerged as promising molecular scaffolds, offering properties such as an enhanced hydrophilicity and reduced toxicity, thereby optimizing bioavailability and pharmacokinetics.

Polysaccharide-based hydrogels and nanoparticles are increasingly utilized for drug delivery, tissue engineering, and wound healing applications [1,2]. The biocompatibility of such scaffolds, particularly hydrogels based on hydroxyethylcellulose mixed with sodium alginate, promotes cell proliferation [3]. Modified polysaccharides with targeted drug delivery capabilities enhance therapeutic efficacies while minimizing side effects, offering personalized treatment options for various medical conditions [4,5]. Biocompatible and biodegradable polysaccharide scaffolds provide a conducive environment for cell growth and tissue regeneration, with applications ranging from bone substitutes to skin grafts [6,7].

Polysaccharides such as pectin, carrageenan, and alginate serve as essential gelling, thickening, and stabilizing agents in food products. Advances in polysaccharide modification and formulation techniques have led to the development of functional ingredients with improved textures, flavor release, prebiotic properties, and shelf stabilities [8,9]. Biodegradable and renewable polysaccharide polymers offer alternatives to conventional petroleum-based plastics, reducing the dependence on non-renewable resources and mitigating plastic pollution. Bio-based composites reinforced with polysaccharide fibers or nanoparticles exhibit impressive mechanical properties and biodegradability, making them suitable for various applications such as in the automotive, construction, and packaging industries [10]. Polysaccharide-based edible films and coatings offer sustainable packaging solutions, extending the shelf life of fresh produce and reducing food waste [11,12].

Polysaccharide-based materials demonstrate promise in environmental remediation efforts, such as water purification and soil stabilization [13,14].

Polysaccharides serve as feedstocks for the production of biofuels and biochemicals through fermentation and enzymatic processes [15,16].

Biopolymer-based membranes and catalysts show promise in energy conversion and storage applications, such as fuel cells and hydrogen production [17]. Polysaccharide-derived carbon materials serve as electrodes in energy storage devices, contributing to the development of sustainable battery and supercapacitor technologies [18].

In conclusion, the recent advancements in the applications of polysaccharides and polysaccharide-based materials hold immense potential for addressing global challenges in food security, healthcare, environmental sustainability, and energy production. Through interdisciplinary research and collaboration, polysaccharides offer innovative solutions not just for today but also pave the way for a more sustainable and resilient future. Continued investment in research and development is essential to unlock the full potential of polysaccharides and accelerate their adoption in various industries.

Given this context, we are pleased to announce the publication of a Special Issue focusing on the current trends and applications of polysaccharide-based materials. We trust that readers will find the articles within this Special Issue insightful, as they delve into the advanced applications and current advancements of polysaccharide materials, offering valuable insights for the benefit of humanity.
